# Measurement of blood pressure in people with atrial fibrillation

**DOI:** 10.1038/s41371-019-0261-4

**Published:** 2019-10-02

**Authors:** Christopher E. Clark, Sinead T. J. McDonagh, Richard J. McManus, on behalf of the Blood Pressure Measurement Working Party of the British and Irish Hypertension Society

**Affiliations:** 1 Primary Care Research Group, Exeter College of Medicine and Health, University of Exeter, Exeter, UK; 2 Nuffield Department of Primary Care Health Sciences, University of Oxford, Oxford, UK

**Keywords:** Diagnosis, Atrial fibrillation, Hypertension

## Executive summary

Current validation protocols for assessing the accuracy of blood pressure monitors exclude people with atrial fibrillation, except in special circumstances [1]. Hypertension guidelines advise manual blood pressure measurement in the presence of arrhythmia. They also promote home and ambulatory monitoring for diagnosis of hypertension, which necessarily requires automated devices [2]. Few studies of automated blood pressure measurement with atrial fibrillation have been undertaken, and none in full accordance with internationally recognised protocols. We recently reported a systematic review and meta-analysis of accuracy of oscillometric devices for blood pressure measurement in atrial fibrillation [3]. The recommendations presented here are based on that review, supplemented by consensus expert opinion of the Blood Pressure Measurement Working Party of the British and Irish Hypertension Society (BIHS).

## Full statement

### Office or clinic blood pressure measurement in atrial fibrillation

Office blood pressure measurements should be carried out using an auscultatory method with a calibrated analogue sphygmomanometer. Current and forthcoming BIHS/National Institute for Health and Care Excellence guidance regarding cuff size, seating and avoidance of other causes of error in blood pressure measurement should be followed [2, 4]. Cuff deflation should be no faster than 2–3 mmHg per second.

Auscultatory methods are recommended due to the lack of evidence for accuracy of most oscillometric devices in the presence of atrial fibrillation [3].

Measurements should be repeated at least three times, regardless of absolute blood pressure, since there is increased intra-person variation in blood pressure with atrial fibrillation. Using an average of these multiple measurements is advised on the basis of expert opinion.

### Home or clinic based automated blood pressure measurement

Due to the absence of evidence for accuracy for most automated monitors designed for home or clinic use, and the increased individual variation in blood pressure with atrial fibrillation, we recommend comparison with multiple auscultatory clinic blood pressure readings for all individuals, when automated devices are used. This may be achieved by taking a sequence of at least three device readings alternating with three auscultatory readings made on the same arm, using a professional analogue device (such as the Accoson Green Light 300).

Limited data do exist to suggest that two monitors, designed for professional use, are accurate in measuring systolic, but not diastolic blood pressure: Philips SureSigns VSi and Welch Allyn Vital Signs 300 devices [5, 6].

For home use, data from one other study suggest that the Tensoval duo control device is accurate in measuring systolic and diastolic blood pressure [7].

The Microlife Watch BPA 100 Plus device may be accurate for systolic but not diastolic blood pressure [8].

### Ambulatory blood pressure measurement

Given the absence of evidence for accuracy of most ambulatory monitors, and the increased individual variation in blood pressure with atrial fibrillation, we recommend comparison of such devices with multiple auscultatory clinic blood pressure readings for all individuals. This may be achieved by taking a sequence of at least three device readings alternating with three auscultatory readings made on the same arm, using a professional analogue device (such as the Accoson Green Light 300).

Limited data obtained by static comparison of oscillometric ambulatory blood pressure monitors with mercury readings suggest accuracy for systolic but not diastolic blood pressures measured by the SpaceLabs 90207 and the A&D TM-2430 devices [9–11].

### Devices with atrial fibrillation detection

Some devices detect pulse irregularity to indicate potentially undiagnosed atrial fibrillation. Such devices are not inherently more accurate in measuring blood pressure with atrial fibrillation, nor can they be assumed to be, in comparison with monitors lacking such technology.

### Automated devices shown to be inaccurate in atrial fibrillation

Based on published evidence, a number of devices have been shown to be inaccurate for blood pressure measurement with atrial fibrillation, and cannot be recommended (Table 1).

**Table 1:** Summary of automated devices studied in measuring blood pressure with atrial fibrillation

Devices with some evidence for accuracy	Devices shown to be inaccurate
Clinic or home measurement	Accutracker 1 [12]
Systolic: Microlife Watch BPA 100 Plus [8]	Copal UA-251 [12]
Systolic: Philips SureSigns VSi [6]	Microlife BP A6 [13]
Systolic: Welch Allyn Vital Signs 300 [5]	Omron HEM 711AC [14]
Systolic and diastolic: Tensoval duo control [7]	Omron HEM-750CP [15]
	Takeda UA-751 [12]
Ambulatory measurement	Welch Allyn 52000 [12]
Systolic: A&D TM-2430 device [9].	
Systolic: SpaceLabs 90207 device [10, 11].	
Diastolic: SpaceLabs 90207 [11]	

## Conclusion

Manual measurement should be used where possible for measuring blood pressure with atrial fibrillation. Limited data exist to suggest that some automated devices can accurately measure systolic, but not diastolic, blood pressure in the presence of atrial fibrillation. Further work is needed to evaluate the many commonly used devices lacking such data. Future comparisons should follow internationally recognised protocols to ensure validity and facilitate comparisons.
