# Correction: Measurement of blood pressure in people with atrial fibrillation

**DOI:** 10.1038/s41371-020-0329-1

**Published:** 2020-03-20

**Authors:** Christopher E. Clark, Sinead T. J. McDonagh, Richard J. McManus, on behalf of the Blood Pressure Measurement Working Party of the British and Irish Hypertension Society

**Affiliations:** 1 Primary Care Research Group, Exeter College of Medicine and Health, University of Exeter, Exeter, UK; 2 Nuffield Department of Primary Care Health Sciences, University of Oxford, Oxford, UK

**Correction to:Journal of Human Hypertension**

10.1038/s41371-019-0261-4

published online 2 October 2019

This article was originally published under Nature Research’s License to Publish, but has now been made available under a [CC BY 4.0] license. The PDF and HTML versions of the article have been modified accordingly.

